# Myotomie par voie transthoracique d'un cas de mégaœsophage géant à l'hôpital du Mali

**DOI:** 10.11604/pamj.2015.21.239.7410

**Published:** 2015-08-04

**Authors:** Seydou Togo, Ouattara Moussa Abdoulaye, Li Xing, Yena Sadio

**Affiliations:** 1 Service de Chirurgie Thoracique, Hôpital du Mali, Bamako, Mali; 2 23^ème^Mission Médicale Chinoise au Mali, Mali

**Keywords:** Mégaoesophage hyper dilaté, milieu rurale, œsocardiomyotomie, voie transthoracique, Hyper dilated megaesophagus, rural environment, cardiomyotomy, transthoracic

## Abstract

Le but de ce travail est de présenter un cas clinique de mégaœsophage « en chaussette » hyper-dilaté occupant presque l'hémithorax droit chez un patient vivant en milieu rurale qui a été pris en charge dans notre centre par une myotomie de Heller par voie transthoracique et décrire les aspects cliniques paracliniques et thérapeutiques. L’œsocardiomyotomie de Heller par voie transthoracique associé à une réduction de la taille de l’œsophage a été réalisé avec la mise en place d'un système anti retour par le biais d'un lambeau diaphragmatique. Le transit œsogastroduodénale, l'endoscopie et le scanner gardent une place importante dans la recherche diagnostique et le choix du traitement.

## Introduction

La forme anatomoclinique du mégaœsophage « en chaussette » peut évoluer vers une dilatation massive en l'absence d'une prise en charge précoce [1, 2]. Malgré les progrès réalisés dans le domaine du diagnostic, le MOI reste un véritable challenge dans les pays sous développés du fait du retard diagnostique et de l'inaccessibilité aux moyens diagnostiques souvent trop couteux. L’œsocardiomyotomie par voie transthoracique associé à la réduction de la taille de l’œsophage est une expérience nouvelle dans notre centre que nous avons réalisée avec un bon résultat. Le but de ce travail est de présenter un cas de mégaœsophage « en chaussette » hyper-dilaté occupant presque l'hémithorax droit chez un patient vivant en milieu rurale qui a été pris en charge dans notre centre par une myotomie de Heller par voie transthoracique et décrire les aspects cliniques paracliniques et thérapeutiques.

## Patient et observation

**Figure 1:**
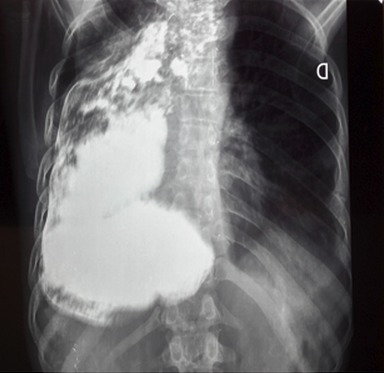
TOGD; megaœsophage «en chaussette» avec une absence d'opacification gastroduodenale

**Figure 2:**
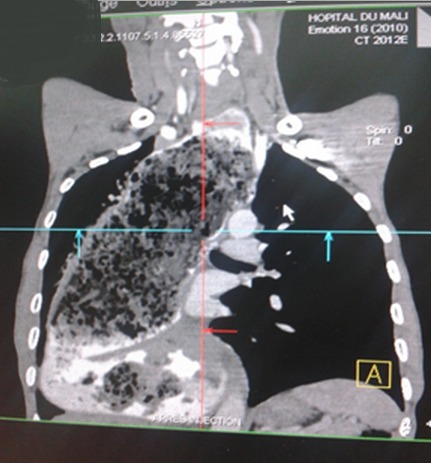
TDM thoracique avec un mégaœsophage très dilaté contenant des débris alimentaires

**Figure 3:**
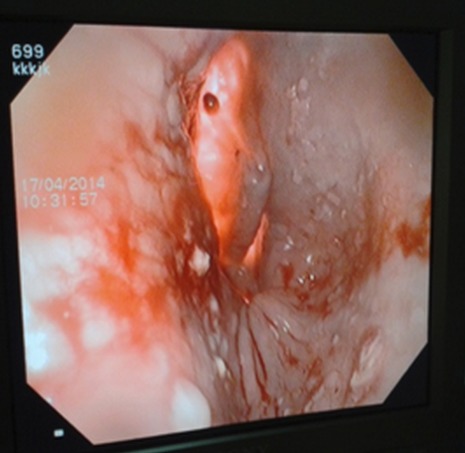
Myotomie sous contrôle endoscopique

**Figure 4:**
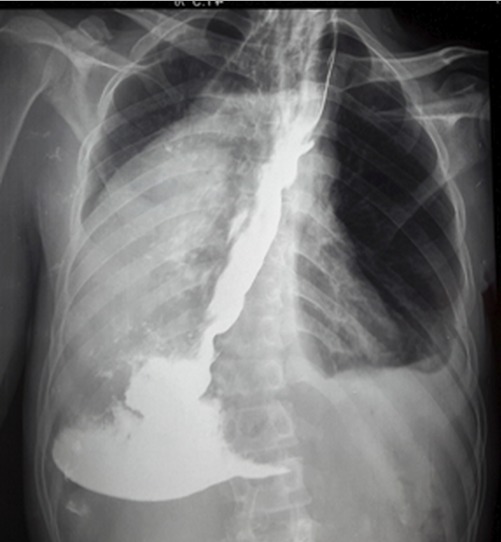
TOGD post opératoire avec réduction du calibre de l’œsophage et opacification gastro-duodénale

Nous rapportons un cas malien de mégaœsophage « en chaussette », très volumineux avec une dilatation massive occupant presque l'hémithorax droit chez un patient âgé de 39 ans, ouvrier résidant en milieu rurale et suivi depuis 19 ans dans un hôpital rurale de la place chez qui les investigations sur la base d'un cliché radiologique réalisé ont permis de conclure à une bronchopneumopathie chronique à germes banals associé à une œsophagite. Le patient présentait une symptomatologie qui remonterai à environ 20 ans marqué par l'apparition de toux productive chronique avec expectoration blanchâtre accentuée surtout après les repas, de douleur thoracique à type de crampe déclenchée par les repas et calmées par les vomissements alimentaires. Une apparition de dysphagie élective aux solides d'installation progressive d’évolution intermittente accompagnée de régurgitation, de dyspnée modérée associée à l'amaigrissement, l'anorexie, et l'asthénie. Plusieurs traitements non spécifiques ont été effectués sans succès. Il est évacué à l'hôpital du Mali avec un état général altéré avec des plis de déshydratation et de dénutrition. L'indice de performance OMS quotté à 2, les murmures vésiculaires étaient diminués au niveau de la base de l'hémithorax droit avec la présence de râles dans les deux champs pulmonaires. L'IMC était à 15,94. A son admission un (transit œso-gastro-duodenal) TOGD réalisé retrouve l’œsophage distendu, hypotonique, dévié à droite avec stase alimentaire et un aspect arrondi du cardia sans opacification gastro duodénale (Figure 1). La Fibroscopie (FOGD) retrouve une achalasie du cardia associé à une gastrite congestive. Il existait une atrophie de l'estomac. La TDM thoracique a permis de visualiser un œsophage distendu, volumineux qui occupait presque l'hémithorax droit avec une stase alimentaire importante (Figure 2). Le diagnostique de mégaœsophage idiopathique « en chaussette » fut retenu. Il existait un situs inversus de l'estomac chez le patient. Le bilan préopératoire retrouve une anémie normocytaire normochrome avec un taux d'hémoglobine à 9,6 g/dl qui fut corrigé avant l'intervention. Nous procédons à une thoracotomie antéro-latérale droit passant par le 6^ème^ espace intercostal, l’œsophage est disséqué, isolé et mis sur laque. Une œso-cardiomyotomie selon Heller est réalisée sur environ 10 cm sur la partie inferieur de l’œsophage thoracique et aussi sur environ 2cm sur le cardia. Le muscle œsophagien était hyper vascularisé. Cette néovascularisation a rendu très difficile la myotomie parce qu'il existait un saignement souvent difficile à contrôler. En raison de la dilatation massive œsophagienne, nous avons été assisté par une endoscopie digestive per-opératoire qui nous a permis d'aspirer les débris alimentaires intra-œsophagiens et de contrôler l'intégrité de la muqueuse afin d'assurer l'efficacité de la myotomie (Figure 3). Une dissection de la muqueuse est réalisée sur les 2/3 en circonférentiel et cela sur toute la longueur de la myotomie. Une plicature de la muqueuse est réalisée sur toute la longueur de la myotomie intrathoracique afin de réduire la taille de l’œsophage. Un lambeau diaphragmatique est prélevé puis suturé en points séparés sur la partie inferieure de la myotomie permettant de constituer une valve anti-retour et d'augmenter la motricité œsophagienne post opératoire. La thoracotomie est fermée sur un drain basi-thoracique mis en siphonage. La reprise alimentaire est autorisée à J.1 post opératoire. Les aliments liquides et semi liquides sont autorisés les premiers jours puis solides de façon progressive. Le patient était sans plainte 1 semaine après l'intervention. Un regain pondéral de 3.5 kgs est obtenu 1 mois après l'intervention. La manométrie œsophagienne n’étant pas disponible, Un TOGD a été réalisé 3 mois en postopératoire et retrouve une bonne opacification gastroduodénale avec une nette diminution du calibre de l’œsophage (Figure 4).

## Discussion

Le taux de succès de la chirurgie du MOI est de 93 à 95%. La chirurgie est le meilleur moyen pour soulager efficacement et durablement les symptômes du MOI tel que le démontrent plusieurs auteurs [3, 4]. C'est l'important retard au diagnostic qui explique les révélations tardives de dilatation massives. L’œsocardiomyotomie transthoracique associé à la réduction de la taille de l’œsophage par plicature de la muqueuse œsophagienne réalisé dans notre cas a permis d'une part de relaxer le sphincter inferieur de l’œsophage et d'autre part de lever l'effet de compression intra thoracique des organes de voisinage. Ceci a permis d'obtenir une amélioration de la fonction cardio- respiratoire et au malade de s'alimenter dans un bref délai. Ce succès thérapeutique est la conséquence d'une approche nouvelle de réduction du volume de l’œsophage, de la réduction de la pression du SIO et le renforcement de la motricité ‘sophagienne après myotomie. L'identification précise de la sous-muqueuse est faite par certaines équipes sous contrôle endoscopique ou en utilisant un ballonnet [5]. Nous avons réalisé dans notre cas la myotomie sous contrôle endoscopique d'autant plus que la paroi de l’œsophage était très dilaté et néovascularisée. Avec l'atrophie gastrique, l’œsophage hyperdilaté et néovascularisée jouait en partie le rôle de l'estomac. Il participait à l'absorption d’éléments nutritifs indispensables à la nutrition du patient. L'adjonction d'un système anti-reflux (SAR) à la myotomie pour prévenir le RGO est soutenue par plusieurs auteurs [1, 6, 7]. Nous avons dans notre cas réalisé un système anti reflux par la mise en place d'un lambeau diaphragmatique pour pouvoir diminuer la tension interne. La contraction de ce lambeau lors des mouvements diaphragmatique améliore la tonicité et la motricité au niveau du bas œsophage permettant de chasser le contenu vers l'estomac. Ceci a permis a notre avis d’éviter le reflux gastro-œsophagien post opératoire d'une part et d'autre part de remédier à l'atonie œsophagienne post opératoire améliorant ainsi la qualité de vie du patient.

## Conclusion

Le retard diagnostique du mégaœsophage idiopathique rencontré surtout en milieu rurale peut souvent conduire à une dilatation monstrueuse de la paroi œsophagienne. Les moyens diagnostiques tels que le TOGD, la fibroscopie et la TDM garde une place très importante dans la recherche diagnostique et la prise en charge chirurgicale. L’œsocardiomyotomie de Heller par voie transthoracique associé à la chirurgie de réduction du volume de l’œsophage est mieux indiquée dans un tel cas. En zone rurale au Mali, la promotion de la télémédecine peut être un outil crédible d'appui à la recherche diagnostique et réduire le délai de prise en charge chirurgicale.
